# Supplementary material for: Femtometer-amplitude imaging of coherent super high frequency vibrations in micromechanical resonators
Source: Nat Commun. 2022 Feb 4;13:694. doi: 10.1038/s41467-022-28223-w (PMC8816924; doi:10.1038/s41467-022-28223-w)
Supplement: Supplementary file 1 — Supplementary Information [file 41467_2022_28223_MOESM1_ESM.docx]

***Supplementary Information***

**Femtometer-amplitude imaging of coherent super high frequency vibrations in micromechanical resonators**

Lei Shao^1,2,*^, Vikrant J. Gokhale^1,3^, Bo Peng^4,5^, Peng-Hui Song^4,5^, Jingjie Cheng^2^, Justin Kuo^6^, Amit Lal^6^, Wen-Ming Zhang^4,5^, and Jason J. Gorman^1,*^

^1^National Institute of Standards and Technology, Gaithersburg, Maryland, USA

^2^University of Michigan-Shanghai Jiao Tong University Joint Institute, Shanghai Jiao Tong University, Shanghai, China

^3^U.S. Naval Research Laboratory, Washington, DC, USA

^4^School of Mechanical Engineering, Shanghai Jiao Tong University, Shanghai, China

^5^State Key Laboratory of Mechanical System and Vibration, Shanghai Jiao Tong University, Shanghai, China

^6^School of Electrical and Computer Engineering, Cornell University, Ithaca, New York, USA

^*^e-mail: lei.shao@sjtu.edu.cn, gorman@nist.gov

1. **Effective wavelength for pulsed laser interferometry**

Two-beam laser interferometers, as commonly used in vibration detection, measure the relative phase, *ϕ*, between the measurement and reference beam paths to determine displacement, *d*, using *d* = *ϕ*∙ *λ*/(4*π*), where *λ* is the laser wavelength. Since ultrafast pulsed lasers have a broad and typically complex optical spectrum as shown in Fig. 1a, unlike single wavelength CW lasers, there is ambiguity in the value of *λ* when using pulsed laser interferometry (PLI). Fig. 1b shows the field auto-correlation for two interfering pulses, consisting of a dense sinusoid enveloped by a decaying function for large delay between the pulses.


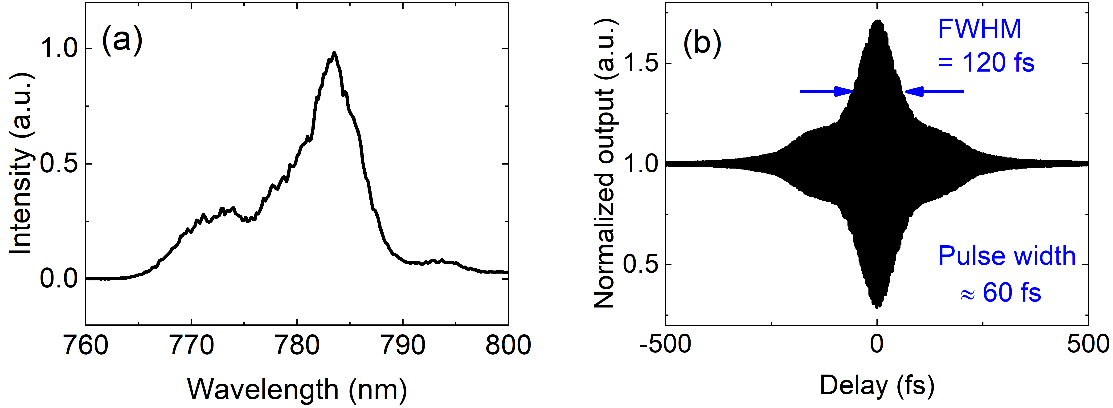


**Fig. 1. Basic characteristics of the ultrafast laser pulses.** (a) Measured optical spectrum shows a broad and asymmetric spectrum unlike that of a single-frequency continuous-wave laser commonly used in interferometry. (b) Field auto-correlation for interfering pulses showing a full width at half maximum (FWHM) of approximately 120 fs.

Recently, we have shown that the fringes for PLI are defined by a single wavelength^1^, as depicted in Fig. 2. However, this effective wavelength, *λ_eff_* , varies significantly as a function of overlap between the interfering pulses due to pulse asymmetry and nonlinear chirp^2^. A Michelson interferometer that combines PLI and continuous-wave laser interferometry (CWLI) has been used to measure the variation in *λ_eff_* as a function of time delay between interfering pulses, as shown in Fig. 3.


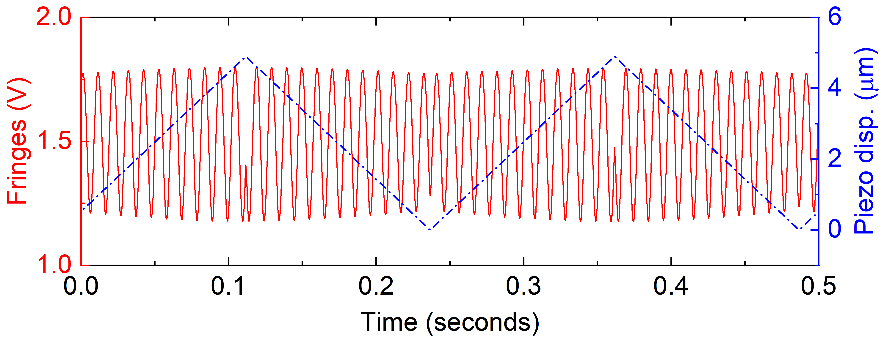


**Fig. 2. Fringes of PLI.** The red curve shows the fringes measured by the slow photodetector while the reference mirror is actuated with a piezoactuator that is driven by a 50:50 triangular wave shown in blue.


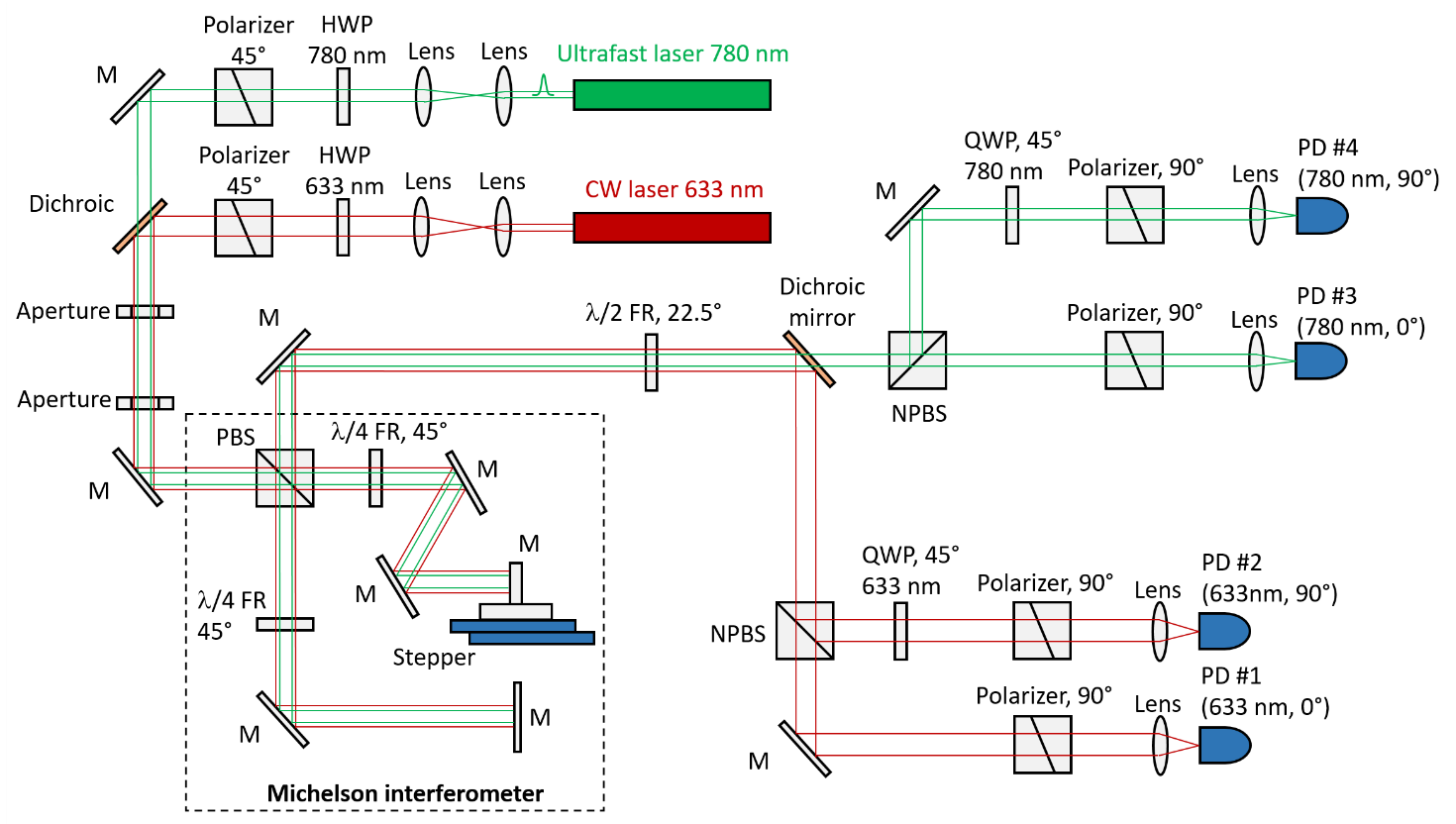


**Fig. 3. Schematic of the setup for characterizing *λ_eff_*.** A Michelson interferometer using pulsed laser interferometry (PLI) and continuous wave laser interferometry (CWLI) are combined for simultaneous measurement with two-quadrature readout for each laser path. HWP: half-wave plate, QWP: quarter-wave plate, M: mirror, PBS: polarizing beam splitter, NPBS: non-polarizing beam splitter, λ/2 FR: half-wave Fresnel rhomb, λ/4 FR: quarter-wave Fresnel rhomb, PD: photodetector.

Using the setup shown in Fig. 3, fringe data from the PLI and CWLI were collected simultaneously while the stepper in the measurement arm moved linearly over 100 μm (nominal step size = 0.01 μm, step dwell time = 0.1 second), which is equivalent to a delay range between the two interfering pulses of approximately +/- 333 fs. The two quadratures from the CWLI provide raw fringe data that is used to calculate the phase angle, *ϕ_C_*, using a well-established data processing procedure^3^. To obtain the displacement, *d*, *ϕ*_C_ is then unwrapped and multiplied by *λ*/(4*π*), where *λ* is the wavelength of the helium-neon laser (i.e., 632.82 nm for the temperature, pressure, and relative humidity during the experiments^4^). Using *λ* = 4π*d*/*ϕ*, *λ_eff_* can then be determined as a function of pulse delay by dividing the CWLI-obtained displacement, *d*, by the PLI-obtained phase, *ϕ_P_*, for each 1 μm increment measured over the 100 μm stepper displacement. The results for *λ_eff_* are shown in Fig. 4 for 10 measurement runs. It is clear that although the variation in *λ_eff_* is quite significant, *λ_eff_* is almost a constant value for near-zero delay between the two interfering pulses. Therefore, there is a single *λ_eff_* that can be used to calculate displacement as long as we keep the delay small while operating the interferometer, which can be achieved by active stabilization.


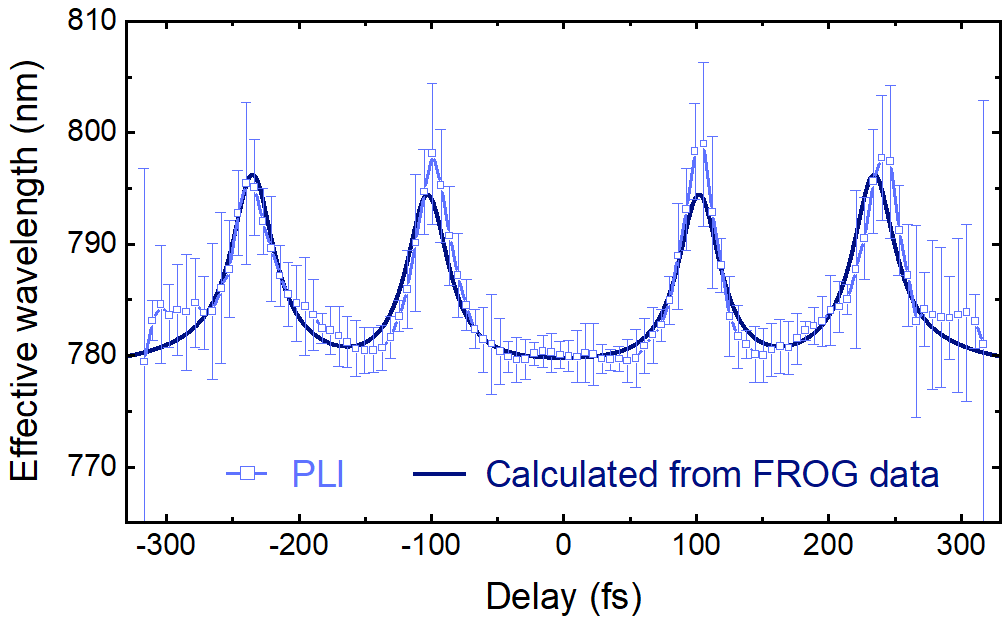


**Fig. 4. *λ_eff_* for pulsed laser interferometry.** *λ_eff_* measured by PLI (in light blue, error bars represent +/- σ), and *λ_eff_* calculated using the experimental pulse characterization data based on the method known as frequency-resolved optical gating (FROG), which yields time-domain intensity and phase of the ultrafast laser pulse (in dark blue)^2^.

In order to obtain the *λ_eff_* for the condition when one interferometer arm is replaced with a sample that is measured through a microscope objective, we ran the above experiment again with the laser pulses in the measurement arm focused on the surface of a bulk acoustic wave resonator (BAW) while in the other arm, the pulses are reflected back from a mirror mounted on a piezoelectric nanopositioner sitting on a stepper stage. This measurement is necessary because the effective wavelength depends on the spectra of the two interfering pulses^2^ and the surface of the BAW and microscope objective will change the reflected pulse spectrum differently than the reference mirror. We experimentally obtained the fringe data by stepping the mirror through the overlapping duration of the two interfering laser pulses, which yields the field auto-correlation data shown in red in Fig. 4. Based on the fringe data with its quadrature, along with fringes obtained simultaneously using a frequency stabilized HeNe laser as described above, we can calculate the *λ_eff_* for performing PLI on the BAW, as shown in blue in Fig. 5. *λ_eff_* is found to be 783.6 nm for near-zero delay.


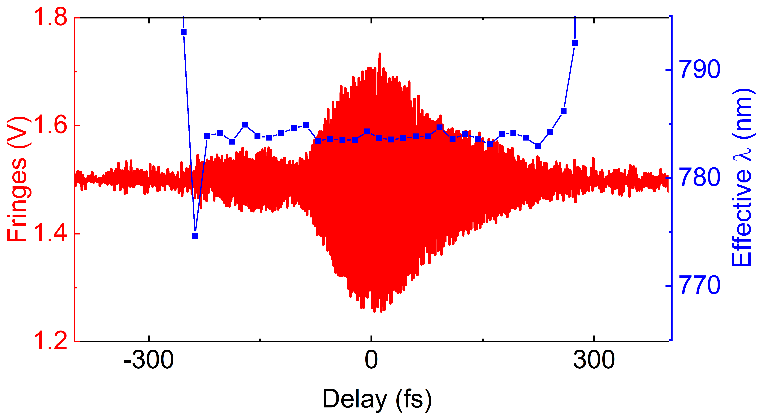


**Fig. 5. Field auto-correlation and *λ_eff_*.** The field-autocorrelation is shown in red while the calculated *λ_eff_* is shown in blue.

1. **Absolute vibrational amplitude and its uncertainty**

The gain factor for converting photodetector voltage to displacement can be accurately measured as the slope at the quadrature point for those fringes with maximum peak-to-peak voltage, $V_{pp}$, in Fig. 2. For each period in the sinusoidal fringe data, the displacement is half of the optical wavelength, which is the *λ_eff_* for PLI. Therefore, the gain factor can be easily found as

| $Gain=2\pi\frac{V_{pp}}{\lambda_{eff}}$, | (S1) |
| --- | --- |

and by this gain factor, the voltage signal obtained using a lock-in amplifier can be converted into absolute displacement. The above approach is a conventional method to obtain the absolute displacement in an SI unit using optical interferometry. The difference is that an effective optical wavelength is used here assuming a fixed delay time for pulsed laser interference, instead of a well-known single wavelength for a CW laser. Thus, the uncertainty of the measured displacement is dominantly determined by the uncertainty in *λ_eff_*, which varies as a function over the delay (or the displacement under test). We have shown in prior work^2^ that the uncertainty in *λ_eff_* is about 10^-4^, which is only a little more than an order of magnitude larger than the wavelength uncertainty for the stabilized helium-neon lasers typically used for CWLI^4^, making it possible to use PLI for accurate displacement measurements.

This uncertainty may be further improved by controlling pressure and temperature, reducing the environmental noise due to acoustics, airflow, and temperature drift, and achieving greater intensity and frequency stability for the pulsed laser. We note that the jitter in the optical frequency components of a typical ultrafast laser ranges from hundreds of hertz to thousands of hertz, which is negligible for these interferometry measurements.

1. **Electrical characterization of the bulk acoustic wave resonator**

Electrical measurement of the bulk acoustic wave resonator (BAW) was carried out using a vector network analyzer (VNA). The BAW is mounted on a PCB with an SMA connector and the signal pads are wire bonded. The measurement was performed at room temperature and under ambient conditions. The device is excited by applying a 10 mW AC input signal from the VNA to the two electrodes and an electrical readout of the S11 parameter is obtained (i.e., reflected power), as shown in Fig. 6. A resonant mode near 2.35 GHz was found based on the S11 plot. This resonant mode is consistent with the first thickness mode detected using the optical method presented in Fig. 2 in the letter. We note that many of the spikes in the magnitude plot in the range of 1.2 GHz to 2.8 GHz are due to high-overtone modes caused by reflections through the thickness of the chip. Due to the limit of our VNA, we could only measure up to 4.5 GHz and thus we could not observe the higher-order modes.


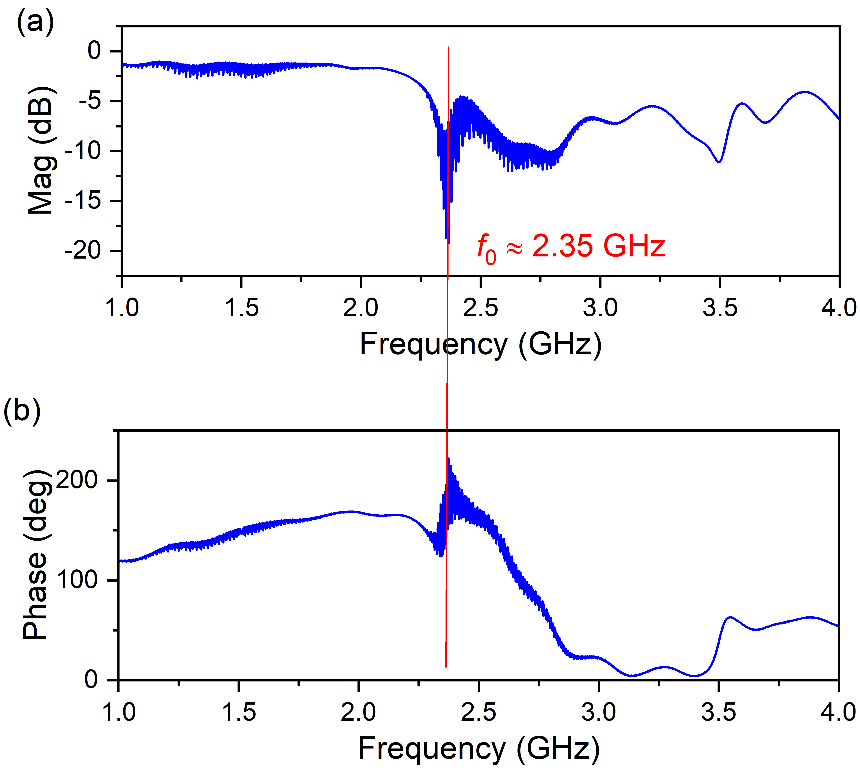


**Fig. 6. Electrical characterization of the BAW.** The magnitude and phase of the S11 parameter of the BAW from 1 GHz to 4 GHz.

Comparing the electrical measurement with the frequency response obtained using stroboscopic optical sampling, it is obvious that the optical method provides better SNR and in addition, provides direct insight into the vibration mode shape and energy dissipation pattern with the spurious modes generated by a superposition of high-order lateral modes. This information would be difficult, if not impossible, to obtain using conventional electrical characterization. Furthermore, we speculate that the small resonant motion at higher-order modes will not show up in an electrical measurement, particularly for the mode at 10.75 GHz, due to the high-frequency feedthrough caused by parasitic capacitances.

1. **Mapping of BAW vibration at more frequencies**

Here we show the vibrational mode profile of the BAW for more frequencies than shown in Fig. 4 in the letter, including both the absolute displacement and phase. It is clearly visible that the spatial period of the horizontal mode gradually decreases as frequency increases until the in-plane resolution of our instrument can no longer distinguish the small periodicity around 3.6 GHz. The laser spot size in the current setup is about 1.9 μm but can be reduced to below 1 μm with some improvements to the optics. By doing this, we expect that lateral modes in the frequency range approaching 7 GHz can be revealed for the presented material system. The effect of the electrical trace as a pathway for acoustic energy leaking to the surrounding structure can be observed at several frequencies, particularly at 2.452 GHz, 2.502 GHz, and 3.602 GHz.





**Fig. 7. Vibration mapping of the BAW.** The vibrational amplitude and phase at multiple frequencies were obtained by scanning the laser spot across the BAW surface.

**Supplementary references**

1. L. Shao, and J.J. Gorman, Pulsed laser interferometry with sub-picometer resolution using quadrature detection, Opt. Exp. **24**, 17459 (2016).
2. L. Shao, J.R. Lawall, and J.J. Gorman, Effect of pulse asymmetry and nonlinear chirp on the accuracy of ultrafast pulsed laser interferometry, Opt. Lett. **42**, 5125 (2017).
3. P. Gregorčič, T. Pozar, and J. Mozina, Opt. Express **17**, 16322 (2009).
4. J. A. Stone, J. E. Decker, P. Gill, P. Juncar, A. Lewis, G. D. Rovera, and M. Viliesid, Metrologia **46**, 11 (2009).
