# Supplementary material for: Femtometer-amplitude imaging of coherent super high frequency vibrations in micromechanical resonators
Source: Nat Commun. 2022 Feb 4;13:694. doi: 10.1038/s41467-022-28223-w (PMC8816924; doi:10.1038/s41467-022-28223-w)
Supplement: Supplementary file 3 — Description of Additional Supplementary Files [file 41467_2022_28223_MOESM3_ESM.docx]

**Description of Additional Supplementary Files:**

**Supplementary Movie 1:** Video demonstrating the reconstructed vibrational mode shape of the piezoelectric thin-film bulk acoustic wave resonator (BAW) excited continuously by a radio-frequency power at 2.352 GHz. The dominant first-order breathing mode shows inphase, out-of-plane motion across the whole surface, with a superposition of lateral, standing wave modes in both of the two horizontal directions.

**Supplementary Movie 2:** Video demonstrating the reconstructed vibrational mode shape of the piezoelectric thin-film bulk acoustic wave resonator (BAW) excited continuously by a radio-frequency power at 6.552 GHz. The third-order breathing mode along the thickness direction is the only visible mode, with a much smaller amplitude compared to that shown in Supplementary Movie 1.

**Supplementary Movie 3:** Video demonstrating the reconstructed vibrational mode shape of the widthextensional silicon bulk acoustic resonator (BAR) excited continuously by a radio-frequency power at 0.983 GHz. It clearly shows the out-of-plane motion of a thirdorder width-extensional resonance due to Poisson coupling, with three anti-nodes spread across the width direction separated by two nodes which stay static entirely.
